# Determination of factors influencing young adults' intention to have COVID-19 vaccine in the Philippines: An integration of Health Belief Model and the Theory of Planned Behavior

**DOI:** 10.1016/j.puhip.2023.100359

**Published:** 2023-02-10

**Authors:** Ardvin Kester S. Ong, Yogi Tri Prasetyo, Fae Coleen Lagura, Rochelle Nicole Ramos, Jose Ma Luis Salazar, Keenan Mark Sigua, Jomy Anne Villas, Reny Nadlifatin, Satria Fadil Persada

**Affiliations:** a School of Industrial Engineering and Engineering Management, Mapúa University, Manila, Philippines. 658 Muralla St., Intramuros, Manila, 1002, Philippines; b International Bachelor Program in Engineering, Yuan Ze University, 135 Yuan-Tung Road, Chung-Li, 32003, Taiwan; c Department of Industrial Engineering and Management, Yuan Ze University, 135 Yuan-Tung Road, Chung-Li, 32003, Taiwan; d Young Innovators Research Center, Mapúa University, Manila, Philippines. 658 Muralla, St., Intramuros, Manila, 1002, Philippines; e Department of Information Systems, Institut Teknologi Sepuluh Nopember, Kampus ITS Sukolilo, Surabaya, 60111, Indonesia; f Entrepreneurship Department, BINUS Business School Undergraduate Program, Bina Nusantara University, Jakarta, 11480, Indonesia

**Keywords:** COVID-19 vaccine, Structural equation modeling, Theory of planned behavior, Health belief model

## Abstract

**Objectives:**

The COVID-19 pandemic continues to increase around the world and businesses and markets across the world significantly decreased. The purpose of this study was to determine the factors that affect the intention to be vaccinated for the COVID-19 vaccine among young Filipino adults by integrating the Health Belief Model and Extended Theory of Planned Behavior.

**Study design:**

A cross-sectional study design was utilized.

**Methods:**

Factors such as understanding of the COVID-19 vaccine, self-efficacy, cues to action, perceived barriers, perceived benefits, perceived side effects, perceived behavioral control, attitude, subjective norm, and intention to be vaccinated were analyzed by utilizing Structural Equation Modeling (SEM).

**Results:**

With 865 young Filipino adults who answered a self-administered survey, it was seen that Understanding of the COVID-19 vaccine has the highest direct significant effect on cues to action, followed by perceived barriers, and perceived benefits. Interestingly, the primary factor was Understanding COVID-19 vaccines which had an indirect significant effect on the intention to get vaccinated. This is because knowing what the vaccine is for, its effects, and the application would lead to the acceptance of the COVID-19 vaccine. Moreover, the impact of being known to have the COVID-19 vaccine would lead to the intention to get vaccinated.

**Conclusions:**

The findings of this study can be utilized especially by the government in developing strategies for encouraging people to take the COVID-19 vaccine. Finally, the model construct of the study can be applied to explore more factors that can affect the intention to be vaccinated with the COVID-19 vaccine and other vaccines people worldwide.

## 1 Introduction

A large part of the world is still in lockdown due to the COVID-19 pandemic and one of the countries that suffered the longest lockdown is the Philippines [1,2]. To which, the COVID-19 vaccines have been developed and are available across the world. However, supplies are not vastly distributed per brand. Considering the Philippines, vaccines are limited based on donations from other countries [3]. The Department of Health [3] has recently updated (March 30, 2022) their records on full vaccine uptake with only 60% of the population, around 66 million out of the 142 Million residents. It was not until the late October of 2021 that the 12-17-year-old population were given authority for vaccination. However, to date, not all teenagers have been fully vaccinated.

As one of the most effective preventive measures in combating the spread of infectious diseases, vaccines are considered a crucial tool to limit the spread of viruses such as the COVID-19 [4,5]. The process of individual immunity by vaccination to herd immunity can help combat infectious diseases [6,7]. Chu and Liu [6] also stated that despite the effective vaccination, many are still reluctant to be vaccinated. This vaccine hesitancy and resistance can be attributed to many factors: the study of which are critical and urgently needed to increase people's acceptance of the COVID-19 vaccine [7,8].

However, people tend to have different perceptions regarding the COVID-19 vaccines [9]. The source of information about vaccines negatively affects the knowledge of people which often leads to confusion [9]. In contrast, Latkin et al. [10] shows that a high percentage of Americans have no intentions to be vaccinated because of racial and gender differences in vaccine intentions, even low trust to the vaccine [10,11].

Despite the increasing number of cases of the COVID-19 in the world, researchers have only focused on the prediction of the spread of the virus [12], preventive measures [13], impact of the COVID-19 [14], and development of vaccines [[15], [16], [17]]. Thus, studying the perception of the COVID-19 vaccine should be explored. Especially in the Philippines, limited to no studies have been conducted regarding the COVID-19 vaccine uptake.

In order to guide health promotion and disease prevention programs, a theoretical model called the Health Belief Model (HBM) could be utilized [18]. The perception towards the current the COVID-19 vaccines could also be assessed using the Theory of Planned Behavior (TPB). Generally, the HBM framework is executed to assess intrapersonal decision-making processes in relevance to a variety of health behaviors such as vaccination and screening [19,20]. Subsequently, TPB is used to accurately predict the intentions and behaviors of an individual, regarding a certain subject matter, by analyzing it through its context, timeframe, motive, and action [22,23]. With this, the application of TPB is evident in different fields of research, including vaccine epidemiology [21,[24], [25], [26], [27], [28], [29], [30], [31], [32], [33]]. With that, there were no studies that utilized the integration of HBM and extended TPB towards the intention to be vaccinated with the COVID-19 vaccine particularly among young adults [[34], [35], [36], [37], [38], [39], [40], [41], [42], [43], [44], [45], [46], [47], [48], [49], [50], [51], [52], [53], [54], [55], [56], [57], [58], [59], [60], [61]].

The purpose of this study was to determine the factors that affect the intention to be vaccinated for the COVID-19 vaccine among young Filipino adults by integrating the Health Belief Model and Extended Theory of Planned Behavior. This study can be used as a strong theoretical foundation for future studies for other vaccines. Finally, the model construct of the study can be applied to explore more factors that can affect the intention to be vaccinated with the COVID-19 vaccine and other vaccines of people around the world.

## 2 Methods

**Fig. 1:**
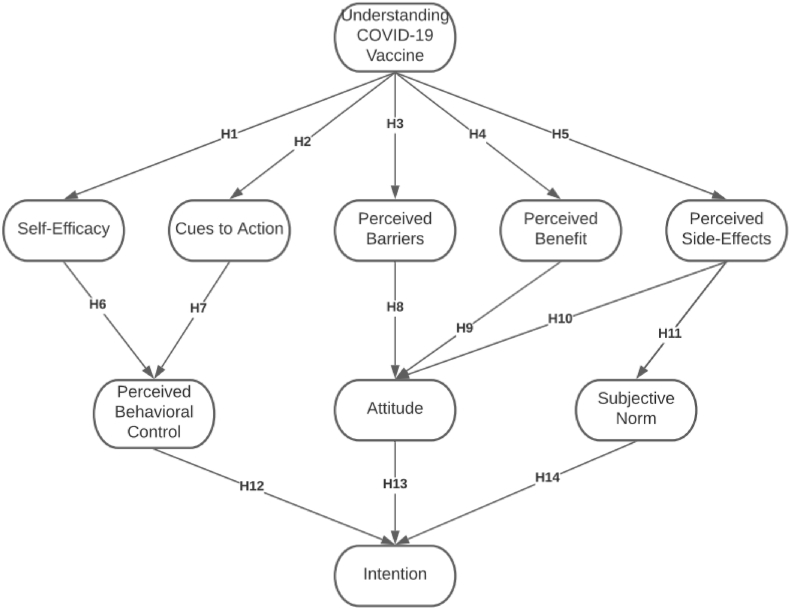
Theoretical framework.

This study was approved by the School of Industrial Engineering and Engineering Management Mapua University Research Ethics Committees and followed the National Ethical Guidelines for Health and Health-Related Research 2017 by the Philippine Health Research Ethics board. Prior to the data collection, all participants were required to fill the consent form that described the purpose of the study and the confidential data of the participants. Fig. 1 demonstrates the framework of this study.

### 2.1 Participants

This study investigated the intention to have the COVID-19 vaccine. Purposive sampling of 865 who voluntarily participated in this study was evaluated. An online self-administered survey was utilized in this study. Through social media platforms, the Google Form was distributed among Filipinos. Following the suggestion of Hair [62], frameworks with 8 or more latent should consider collecting 500 or more data to represent the intendent research upon using SEM. Represented in Table 1 are the descriptive statistics of the respondents.

**Table 1:** Participant characteristics (n = 865).

Characteristics	Category	n	%
Gender	Male	414	47.9
	Female	437	50.5
	Other	14	1.6
Age	15–24 years old	837	96.8
	25–34 years old	28	3.2
Education	Elementary graduate	8	0.90
	Junior high school graduate	516	59.7
	Senior high school graduate	299	34.6
	Technical – Vocation Graduate	2	0.20
	College Graduate	37	4.30
	Master Graduate	3	0.30
Monthly Salary/Allowance	Less than 15,000	741	85.7
	15,000–30,000	56	6.50
	30,000–45,000	24	2.80
	45,000–60,000	16	1.80
	60,000–75,000	8	0.90
	More than 75,000	20	2.30
Religion	Roman Catholic	745	86.1
	Islam	5	0.60
	Hinduism	0	0.00
	Buddhism	3	0.30
	Atheists or Agnostics	38	4.40
	Others	74	8.60
Location	Region I	2	0.20
	Region II	15	1.70
	Region III	9	1.00
	Region IV-A	112	12.9
	Region IV-B	162	18.7
	Region V	11	1.30
	CAR	10	1.20
	NCR	497	57.5
	Region VI	6	0.70
	Region VII	6	0.70
	Region VIII	19	2.20
	Region IX	6	0.70
	Region X	3	0.30
	Region XI	3	0.30
	Region XII	3	0.30
	Region XIII	1	0.10
	BARMM	0	0.00

### 2.2 Questionnaire

This study is one of the first studies that integrate these 2 theories and represented in Table 2 are the constructs utilized for this study. There was a total of 11 sections for the online survey questionnaire divided into demographics, understanding the COVID-19 vaccine, self-efficacy, cues to action, perceived barriers, perceived benefits, perceived side effects, perceived behavioral control, acceptance, subjective norm, and intention. A 5-point Likert Scale was utilized to evaluate the constructs of this study [[64], [65], [66], [67], [68], [69], [70]].

**Table 2:** Construct and measurement items.

Variable	Code	Constructs	Reference
Understanding of the COVID-19 Vaccine	U1	I understand the basic information about the COVID-19 Vaccine.	
	U2	I understand the effects of the COVID-19 Vaccination.	
	U3	I understand the developmental process of the current COVID-19 Vaccines.	
	U4	I am familiar with the current COVID-19 vaccines.	
	U5	I understand the applicability of the differences of the current COVID-19 vaccines.	
Self- Efficacy	S1	I am confident that I will obtain COVID-19 vaccines.	[74]
	S2	Getting vaccinated to prevent COVID-19 is convenient.	[14]
	S3	It will be easy for me to get the vaccines to protect myself from COVID-19.	[14]
	S4	Despite setbacks, I will still pursue getting COVID-19 vaccines.	
	S5	Despite the presented side effects, I am still confident in getting COVID-19 vaccines.	
Cues to Action	C1	The presence of news of COVID-19 vaccine made me want to get the COVID-19 vaccine.	[36]
	C2	The abundance of my vaccinated friends makes me want to get the COVID-19 vaccine.	
	C3	It is my responsibility to get the COVID-19 vaccine.	
	C4	The number of COVID-19 cases makes me want to get the COVID-19 vaccine.	
	C5	The effectiveness of the vaccine convinced me to be vaccinated.	
Perceived Barriers	P1	I am well-informed on the COVID-19 vaccines (side effects, benefits, costs)	
	P2	There is enough information available about the COVID-19 vaccine.	[62]
	P3	I am not afraid of the COVID-19 vaccine.	
	P4	I can afford the COVID-19 vaccine.	
	P5	I believe that the COVID-19 vaccine does more good than harm.	[36]
Perceived Benefits	PB1	Being vaccinated makes me feel safe from COVID-19.	
	PB2	If I get the vaccines, I will be less likely to have severe effect on COVID-19.	[14]
	PB3	I believe that the COVID-19 vaccines are effective in preventing myself in being infected by the virus.	[62]
	PB4	Having myself vaccinated against COVID-19 is beneficial for myself and the health of others in my community.	[14]
	PB5	I believe that the COVID-19 vaccination is an important tool to stop the pandemic.	
Perceived Side Effects	PS1	The side effects of the vaccine make me worried about having one.	
	PS2	The side effects of the current COVID-19 vaccines affects my decisions on getting vaccinated.	
	PS3	I think the vaccine might have too many dangerous side effects.	[75]
	PS4	I think that the COVID-19 vaccine will result to short-term side effects.	[76]
	PS5	I think the COVID-19 vaccine will cause further health problems.	[76]
Perceived Behavioral Control	PBC1	I believe that getting vaccinated can improve the current situation of our society.	[77]
	PBC2	I will feel safe when I get vaccinated.	[78]
	PBC3	Getting vaccinated will improve my confidence on my day-to-day life during the pandemic.	
	PBC4	I believe in my ability to cope up with the side effects of COVID-19 vaccination	
	PBC5	I believe that whether or not I get vaccinated for COVID-19 is entirely up to me	
Acceptance	A1	I do not worry about the COVID19	
	A2	I do not feel stressed whenever I think that I can be positive for COVID-19.	
	A3	I am not afraid that one of my family and friends can be positive for COVID-19	
	A4	I am not afraid that one of my family and friends can die because of COVID-19	
	A5	I think that being vaccinated for the COVID-19 is a responsibility	
Subjective Norm	SN1	I think that my relatives want me to get vaccinated.	
	SN2	I think that my friends want me to get vaccinated.	
	SN3	Most people who are important to me will get vaccinated for COVID-19.	[14]
	SN4	If people around me seeks information for COVID-19 vaccine, I will do the same thing.	
	SN5	My family and friends expect me to be vaccinated.	
Intention	I1	I will get the COVID-19 vaccine immediately when it is available.	[27]
	I2	I would get vaccinated if a physician offered me COVID-19 vaccines.	[14]
	I3	I will encourage my family and friends to be vaccinated.	[79]
	I4	If a COVID-19 vaccine is proven safe and effective, and is available, I will take it.	[80]
	I5	I will most likely get vaccinated if a healthcare provider strongly recommends it.	[81]

### 2.3 Structural Equation Modeling

SPSS 25 and AMOS23 were utilized to derive the SEM in analyzing the intention to get the COVID-19 vaccine. Hair [62] and Kiraz et al. [68] explained how different exogeneous and endogenous latent variables could be analyzed simultaneously using SEM. Moreover, the strength of SEM highlights the acceptability of the models being utilized in the study [63].

## 3 Results

**Fig. 2:**
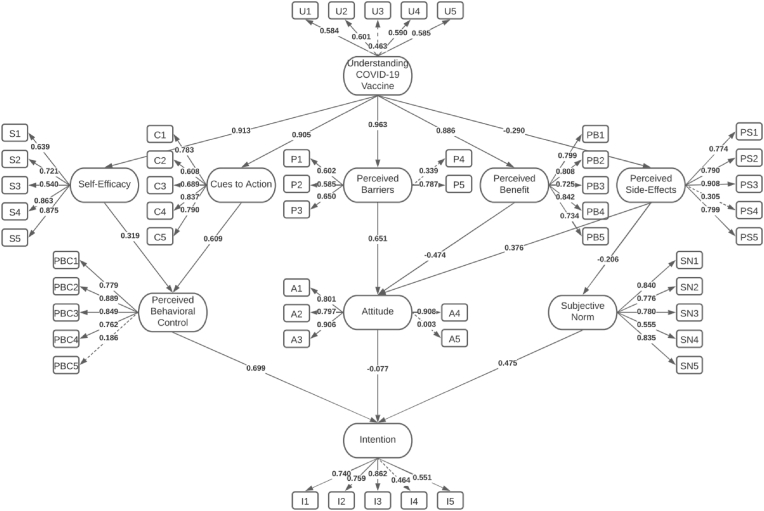
Initial SEM for the intention to receive the COVID-19 vaccine.

Represented in Fig. 2 is the initial SEM model for the intention to have the COVID-19 vaccine uptake. As recommended by Hair [62], insignificant indicators with factor loadings lower than 0.50 may be removed to enhance the model fit. Factor loadings represent the affecting weights for the unobserved latent variables considered in this study [71].

**Fig. 3:**
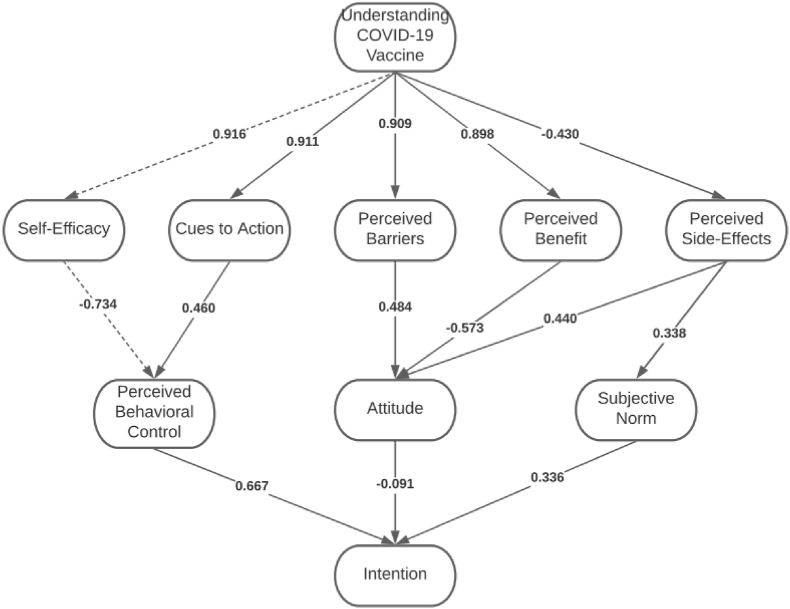
Final SEM for the intention to receive the COVID-19 vaccine.

Fig. 3 represents the final SEM model for intention to have the COVID-19 vaccine. After the removal of insignificant indicators, it was seen that understanding on self-efficacy (S) (p-value = 0.101) and perceived behavior control (PBC) (p-value = 0.940) have insignificant relationship with (p-values greater than 0.05) [62]. Moreover, the values between two latent variables are measured using the beta (β) coefficient which represents the correlation of the direct effect. The higher the value, the more highly influential the effect is on the overall model [71]. Moreover, presented in Table 3 are the descriptive statistics of the indicators utilized in this study.

**Table 3:** Indicators: statistical analysis.

Variable	Item	Mean	SD	Factor Loading	
				Initial	Final
Understanding of the COVID-19 Vaccine	U1	4.136	0.804	0.584	0.703
	U2	4.038	0.876	0.601	0.512
	U3	3.808	0.984	0.463	–
	U4	4.133	0.816	0.590	0.633
	U5	3.886	0.943	0.585	0.659
Self- Efficacy	S1	3.376	1.128	0.639	0.638
	S2	4.028	0.957	0.721	0.719
	S3	3.399	1.097	0.540	0.532
	S4	3.658	1.074	0.863	0.875
	S5	3.577	1.084	0.875	0.878
Cues to Action	C1	3.573	1.084	0.783	0.762
	C2	3.108	1.138	0.608	0.576
	C3	3.847	1.051	0.689	0.675
	C4	3.979	1.053	0.837	0.823
	C5	3.602	1.075	0.790	0.766
Perceived Barriers	P1	3.879	1.023	0.602	0.542
	P2	3.629	1.005	0.585	0.558
	P3	3.444	1.208	0.650	0.646
	P4	3.469	1.125	0.339	–
	P5	3.828	0.942	0.787	0.808
Perceived Benefits	PB1	3.834	1.044	0.799	0.838
	PB2	3.951	0.944	0.808	0.799
	PB3	3.784	1.010	0.725	0.717
	PB4	4.157	0.911	0.842	0.848
	PB5	4.210	1.137	0.734	0.718
Perceived Side Effects	PS1	3.493	1.191	0.774	0.587
	PS2	3.476	1.193	0.790	0.526
	PS3	3.066	1.185	0.908	0.860
	PS4	3.666	0.917	0.305	–
	PS5	2.825	1.137	0.799	0.886
Perceived Behavioural Control	PBC1	4.162	0.896	0.779	0.779
	PBC2	3.842	1.007	0.889	0.862
	PBC3	3.837	1.009	0.849	0.810
	PBC4	3.832	1.012	0.762	0.759
	PBC5	4.020	1.004	0.186	–
Acceptance	A1	1.988	1.276	0.801	0.832
	A2	2.221	1.349	0.797	0.859
	A3	1.975	1.297	0.906	0.831
	A4	1.816	1.251	0.908	0.868
	A5	3.982	1.024	0.003	–
Subjective Norm	SN1	3.639	1.187	0.840	0.808
	SN2	3.637	1.142	0.776	0.821
	SN3	3.763	1.062	0.780	0.791
	SN4	4.017	0.892	0.555	0.526
	SN5	3.579	1.178	0.835	0.862
Intention	I1	3.576	1.159	0.740	0.747
	I2	3.747	1.065	0.759	0.739
	I3	3.883	1.033	0.862	0.869
	I4	4.439	0.849	0.464	–
	I5	4.260	0.941	0.551	0.586

The composite reliability of the constructs was calculated to determine the overall consistency and validity (Table 4). Hair [62] and Chuenyindee et al. [72] indicated that values greater than or equal to 0.700 for Cronbach's alpha and Composite Reliability for SEM would dictate internal consistency and validity. Moreover, presented in Table 5 is the parameters to measure the model fit for the SEM. Weston and Gore [71] explained how the GFI values represent the absolute fit of the constructs and how it represents a direct assessment of the observed data AGFI is utilized to measure the increase of the GFI for less restricted model [73].

**Table 4:** Composite reliability.

Factor	Cronbach's α	Average Variance Extracted (AVE)	Composite Reliability (CR)
Understanding of the COVID-19 vaccine	0.850	0.398	0.723
Self-Efficacy	0.853	0.549	0.855
Cues to Action	0.864	0.526	0.846
Perceived Barriers	0.746	0.419	0.737
Perceived Benefits	0.886	0.618	0.889
Perceived Side Effects	0.891	0.536	0.815
Perceived Behavioural Control	0.895	0.646	0.879
Acceptance	0.817	0.719	0.911
Subjective Norm	0.872	0.594	0.877
Intention	0.855	0.551	0.828

**Table 5:** Model fit.

Goodness of fit measures of SEM	Parameter Estimates	Minimum cut-off	Suggested by
Incremental Fit Index (IFI)	0.848	>0.80	Gefen et al. (2000)
Tucker Lewis Index (TLI)	0.833	>0.80	Gefen et al. (2000)
Comparative Fit Index (CFI)	0.847	>0.80	Gefen et al. (2000)
Goodness of Fit Index (GFI)	0.811	>0.80	Gefen et al. (2000)
Adjusted Goodness of Fit Index (AGFI)	0.819	>0.80	Gefen et al. (2000)
Root Mean Square Error (RMSEA)	0.069	<0.07	Steiger (2007)

In addition, IFI measures the small number of sample bias. To which, it measures how likely the model would fit even with small number of sample size [73]. On the other hand, CFI measures the improvement of the fit of the model for it to be accepted [71]. Lastly, Hsu [74] explained how TLI measures the covariance within group functions. As stated by Gefen et al. [75], values for TLI, IFI, CFI, GFI, AGFI, with values greater than 0.800 and RMSEA value less than 0.07 [[76], [77], [78]] are a good fit. Represented in Table 6 are the direct, indirect, and total effects of the latent variables together with their p-values.

**Table 6:** Direct, indirect, and total effects.

No.	Variable^a^	Direct Effect	P-Value	Indirect Effect	P-Value	Total Effect	P-Value
1	U→P	0.909	0.005	–	–	0.909	0.005
2	U→C	0.911	0.019	–	–	0.911	0.019
3	U→PB	0.898	0.002	–	–	0.898	0.002
4	U→PS	−0.430	0.009	–	–	−0.430	0.009
5	U→SN	–	–	0.046	0.011	0.046	0.011
6	PS→SN	−0.338	0.012	–	–	−0.338	0.012
7	P→A	0.484	0.021	–	–	0.484	0.021
8	C→PBC	0.460	0.020	–	–	0.460	0.020
9	PB→A	−0.573	0.018	–	–	−0.573	0.018
10	PS→A	0.440	0.019	–	–	0.440	0.019
11	SN→I	0.336	0.014	–	–	0.336	0.014
12	A→I	−0.091	0.025	–	–	−0.091	0.025
13	PBC→I	0.667	0.009	–	–	0.667	0.009
14	U→PBC	–	–	0.866	0.023	0.866	0.023
15	U→I	–	–	0.639	0.020	0.639	0.020
16	P→I	–	–	−0.065	0.021	−0.065	0.021
17	C→I	–	–	0.359	0.023	0.359	0.023
18	PB→I	–	–	0.005	0.016	0.005	0.016
19	PS→I	–	–	−0.190	0.009	−0.190	0.009

a Variable codes: A, attitude; C, cues for action; I, intention P, perceived barriers; PB, perceived benefits; PBC, perceived behavioural control; PS, perceived side effects; SN, subjective norm; U, understanding.

Understanding of the COVID-19 vaccine had the highest significant direct effects on Cues to Action (β: 0.911; p = 0.019) followed by Perceived Barriers (β: 0.909 and p = 0.005). This suggests that comprehensive knowledge about the COVID-19 vaccine triggers an individual to act and accept vaccination. In addition, Understanding negatively affected the Perceived Side Effects (β = −0.430; p = 0.009). In a study by Teitler-Regev et al. [79], knowledge affects the perceptions towards the vaccine intentions. Similarly, Xie et al. [80] stated that knowledge is a determinant of the effectiveness of a preventive measure. With the said studies, it further proved that knowledge towards a subject matter is a strong determinant of perception and intention. Such cues linked to the COVID-19 consist of direct the COVID-19 experience, health status of family members, and media recommendations [77].

The SEM results suggest that understanding had significant direct effects on Perceived Benefits (β: 0.898; p = 0.002). Wallston et al. [42] posits that perceived benefits provided by Healthcare Information Technology (HIT) are directly influenced by employee factors. This implies that full knowledge about the COVID-19 virus, particularly the biology of the virus, would significantly affect perceived benefits. The relationship between the latent is evident in numerous studies of different health contexts [[81], [82], [83], [84]]. In health belief, Tola et al. [82] stated that lack of knowledge on a specific disease treatment can cause non-adherent behaviors towards disease treatment. In public health, Hwang et al. [83] stated that the degree of knowledge determines the perception and behavior of an individual.

In addition, Perceived Barriers was found to positively and directly affect attitude (β: 0.484 and p = 0.021), and Cues to Action was revealed to have a significant positive direct effect on PBC (β: 0.460 and p = 0.020). Being well-informed, confident, and the affordability of the vaccine is a necessary factor that greatly affects the attitude of people towards it. This also means that having higher perceived barriers towards the vaccine, the lower the possibility for them to accept it. It is supported by the study of Wong et al. [34] which states that perceived barriers are one of the key factors that influence the acceptance of people to the vaccine. It is also stated that many perceived barriers can cause to a negative attitude and refusal of the vaccine [30,42,53,[85], [86], [87]].

Interestingly, Perceived Side Effects was also found to significantly and negatively affect Subjective Norm (β: 0.338 and p = 0.012). Moreover, there is a negative direct effect of Perceived Benefits towards attitude (β: 0.573 and p = 0.018). Based from the construct, some people believed that the COVID-19 vaccine will cause further health problems which affects their decision on vaccine uptake and increases their worry about the vaccine. This is supported by several studies [58,83], wherein the stronger the information dissemination regarding the side effects of vaccines to the public, the more it results in vaccine hesitancy on most of the population.

Contrary to the negative direct results of Perceived Benefits towards Attitude, Perceived Side Effects had a significant positive direct effect towards Attitude (β = 0.440 and p = 0.019). This finding shows that despite the setbacks of the side effects of the COVID-19 vaccine, people believe that the side effects are not dangerous, short-term, and will not cause further health problems. Supporting this result, Kadali et al. [84] showed findings on the BNT1622b2 mRNA vaccine that majority of their participants are eager to take the vaccine as majority of the participants can continue daily activities while few only experienced short-term side effects.

In relation, Subjective Norm was also found to have a significant positive direct effect on Intention (β: 0.336 and p = 0.014). It shows other people, such as friends and relatives, affect their intention to get vaccinated with the COVID-19 vaccine. Participants believed that seeking information and expectation of family and friends is vital in the decision making their intention for vaccination. Subsequently, Attitude is found to be a significant factor in affecting Intention. However, it shows a negative direct effect (β: 0.091 and p = 0.025). The results show that Perceived Benefits is the main reason why attitude has a negative direct effect towards Intention. It is supported by Chu and Liu [6] which stated that benefits of being vaccinated is not enough motivation. In contrasts with the study of Barlett [49], the result showed that attitude determines intention and behavior of people towards the vaccine due to different countries and beliefs.

Lastly, the findings revealed that PBC positively and directly affects Intention (β: 0.667 and p = 0.009). This relationship implies that hindrances and difficulties that one might face before, during, and after the vaccination is a determinant of receiving the COVID-19 vaccines. To further elaborate, several studies [60,88,89] provided findings that support the relationship between the said latent. Each of their studies dealt with individual's perceptions towards vaccines against a different type of disease, revealing similar results of PBC as a strong predictor of vaccine intention.

## 4 Discussion

The novelty of the current the COVID-19 vaccines draws different negative perceptions. Karlsson et al. [55] even stated that the perceived risk towards the COVID-19 vaccines outweighs the perceived risk of the disease itself. Therefore, it is vital to trace the root cause of these negative perceptions for health authorities can provide a solution. It is not just on the COVID-19 virus as a threat to public health but also on the suppositions. With its unfamiliarity and newness, there is still a lack of research on the COVID-19 vaccines, hindering the government and the public health authorities to be knowledgeable on the different perceptions.

It could be seen from the study that integrating both TPB and HBM holistically measured the intention to have the vaccine uptake. Usual studies have considered individual framework to compare results of vaccine hesitancy. Hossain et al. [90] used TPB, HBM, and the 5C antecedents separately to compare the highest variance among the COVID-19 vaccine hesitancy. Utilizing multiple linear regression, their study presented TPB had the highest predictive power, followed by 5C, and HBM. In addition, Shmueli [91] considered grouping factors under TPB and HBM for evaluating intention to receive the COVID-19 vaccines in Israel utilizing a hierarchical logistic regression. Their study showed no causal relationship was drawn due to the methodology used, similar to the study of Patwary et al. [92]. Lastly, Richie et al. [93] considered studies that utilized HBM and TPB separately using meta-analysis. They explained how most studies have considered the individual latent separately, with no consistency on the effectiveness of the models. Thus, this study was able to determine holistically the intention to have the COVID-19 vaccine uptake due to the integration of both HBM and TPB.

### 4.1 Research implications

Out study indicated that understanding the COVID-19 vaccine was seen to be the factor that would drive the people's intention to uptake the vaccine. Based from the results of the study, knowing what the vaccine is for, its effects, and the application would lead to the acceptance of the COVID-19 vaccine. Therefore, this key finding could help government officials in promoting the COVID-19 vaccine uptake [94]. Moreover, the World Health Organization and the Central for Disease Control and Prevention should highlight the importance, benefits, and side-effects of up-taking the COVID-19 vaccine. This would pave a way for better understanding and knowledge towards accepting the COVID-19 vaccine and would lead to the intention to be vaccinated. The findings of this study can be utilized to form strategies in order to attain herd immunity. Attaining herd immunity would make the country go one step further in returning to what was known as the normal state.

### 4.2 Limitations

This study was conducted during the COVID-19 lockdown. With that, this study considered several limitations. First, this study was conducted by distributing questionnaires online. In line with this, majority of the respondents were within the range of 15–24 years old (96.8%). The study tried to cover several age groups, however, since social media platforms were utilized, majority are of the young age group. Second, with the progressive development of the COVID-19 vaccines, it is notable that the findings of this study are limited to the current situation of the pandemic. Additionally, there might be a change in the perceptions of an individual as the vaccines are continuously being improved and as the population of individuals who receive vaccines grow. Lastly, the study only considered perception to get vaccinated. It is also recommended to consider consumer preference on the type of vaccine using conjoint analysis approach.

## 5 Conclusions

The COVID-19 pandemic continuous to increase around the world, therefore, the need for COVID-19 vaccination should be explored. The purpose of this study was to determine the factors that affect the intention to be vaccinated of COVID-19 vaccine among Filipino young adults by integrating the Health Belief Model and Extended Theory of Planned Behavior. With 865 young Filipino adults who answered a self-administered survey, it was seen that Understanding of the COVID-19 vaccine has the highest direct significant effect on cues to action, followed by perceived barriers, and perceived benefits. Interestingly, the primary factor was Understanding COVID-19 vaccines which had an indirect significant effect on the intention to get vaccinated. This is because knowing what the vaccine is for, its effects, and the application would lead to the acceptance of the COVID-19 vaccine. Moreover, the impact of being known to have the COVID-19 vaccine would lead to the intention to get vaccinated.

This study is one of the first studies that integrated HBM and ETPB for analyzing the intention to be vaccinated of COVID-19 vaccine. The findings of this study can be utilized in developing strategies for encouraging people to take the COVID-19 vaccine. In addition, the model construct of the study can be applied to explore more factors that can affect the intention to be vaccinated with COVID-19 vaccine of the people all around the world.

## Funding

This research was funded by Mapúa University Directed Research for Innovation and Value Enhancement (DRIVE).

## Institutional review board statement

This study was approved by Mapua University Research Ethics Committees and followed the National Ethical Guidelines for Health and Health-Related Research 2017 by the Philippine Health Research Ethics board.

## Informed consent statement

Informed consent was obtained from all subjects involved in the study.

## Conflicts of interest

The authors declare no conflict of interest.

## Declaration of competing interest

The authors declare that they have no known competing financial interests or personal relationships that could have appeared to influence the work reported in this paper.
